# The burden of non‐SARS‐CoV2 viral lower respiratory tract infections in hospitalized children in Barcelona (Spain): A long‐term, clinical, epidemiologic and economic study

**DOI:** 10.1111/irv.13085

**Published:** 2022-12-20

**Authors:** Jorgina Vila, Esther Lera, Cristina Andrés, Maria Piñana, Victoria Rello‐Saltor, Marc Tobeña‐Rué, Joan Balcells, Zaira Benítez‐Díaz, Marta Beatriz Aller, Rosario Muñoz, Ana Vázquez, Carlos Rodrigo, Pere Soler‐Palacín, Andrés Antón

**Affiliations:** ^1^ Paediatric Hospital Medicine, Department of Paediatrics Hospital Universitari Vall d'Hebron Barcelona Spain; ^2^ Department of Paediatrics, Obstetrics and Gynecology, Preventive Medicine and Public Health. Faculty of Medicine Universitat Autònoma de Barcelona Barcelona Spain; ^3^ Infection in Immunocompromised Paediatric Patients Vall d'Hebron Research Institute Barcelona Spain; ^4^ Paediatric Emergency Unit, Department of Paediatrics Hospital Universitari Vall d'Hebron Barcelona Spain; ^5^ Respiratory Viruses Unit, Virology Section, Microbiology Department Hospital Universitari Vall d'Hebron Barcelona Spain; ^6^ Paediatric Critical Care Unit, Department of Paediatrics Hospital Universitari Vall d'Hebron Barcelona Spain; ^7^ Department of Information Systems and Decision Support Hospital Universitari Vall d'Hebron Barcelona Spain; ^8^ Health Services Research Group Vall d'Hebron Research Institute Barcelona Spain; ^9^ Department of Applied Statistics Universitat Autònoma de Barcelona Barcelona Spain; ^10^ Department of Paediatrics Hospital Universitari Germans Trias i Pujol Barcelona Spain; ^11^ Germans Trias i Pujol Research Institute Barcelona Spain; ^12^ Paediatric Infectious Diseases and Immunodeficiencies Unit, Department of Paediatrics Hospital Universitari Vall d'Hebron Barcelona Spain

**Keywords:** cost of illness, global burden of disease, hospitalization, respiratory tract infections, viruses

## Abstract

**Background:**

Viral lower respiratory tract infections (LRTI) are the leading cause of hospitalization in children. In Catalonia (Spain), information is scarce about the burden of viral LRTIs in paediatric hospitalizations. The aim of this study is to describe epidemiological, clinical, virological and economic features of paediatric hospitalizations due to viral LRTI.

**Methods:**

From October 2012 to December 2020, children aged <16 years admitted to a tertiary paediatric hospital in Catalonia (Spain) with confirmed viral LRTI were included in the study. Virus seasonality, prevalence, age and sex distribution, clinical characteristics, hospital costs and bed occupancy rates were determined.

**Results:**

A total of 3,325 children were included (57.17% male, 9.44% with comorbidities) accounting for 4056 hospitalizations (32.47% ≤ 12 months): 53.87% with wheezing/asthma, 37.85% with bronchiolitis and 8.28% with pneumonia. The most common virus was respiratory syncytial virus (RSV) (52.59%). Influenza A was associated with pneumonia (odds ratio [OR] 7.75) and caused longer hospitalizations (7 ± 31.58 days), while RSV was associated with bronchiolitis (OR 6.62) and was the most frequent reason for admission to the paediatric intensive care unit (PICU) (11.23%) and for respiratory support (78.76%). Male sex, age ≤12 months, chronic conditions and bronchiolitis significantly increased the odds of PICU admission. From October to May, viral LRTIs accounted for 12.36% of overall hospital bed days. The total hospitalization cost during the study period was €16,603,415.

**Conclusions:**

Viral LRTIs are an important cause of morbidity, hospitalization and PICU admission in children. The clinical burden is associated with significant bed occupancy and health‐care costs, especially during seasonal periods.

## 1 INTRODUCTION

Viral lower respiratory tract infections (LRTI) are an important cause of morbimortality and hospital admissions in children, especially in the population aged under five years.^1^ The most common LRTIs in children are bronchiolitis, community acquired pneumonia (CAP), wheezing and asthma exacerbation.^2^, ^3^, ^4^

Bronchiolitis is a viral infection with an annual incidence of 5.2/100 in children under six months of age in Spain, and an annual hospitalization incidence of 2.1 cases/100 children per year.^2^ It is responsible for up to 13.5% of paediatric intensive care unit (PICU) admissions during the winter months.^5^ The main etiological agent of bronchiolitis is the respiratory syncytial virus (RSV), followed by the rhinovirus (RV).^5^ CAP is also a major cause of illness in children in developed countries. A virus is identified in 43–67% of cases of CAP, with RV, RSV, and influenza viruses being the main culprits worldwide.^3^ Finally, 30–50% of children suffer one episode of wheezing before school age, and 30–40% present recurrent episodes. Asthma is the leading cause of chronic disease worldwide, with a prevalence of 10% in Western Europe.^4^ Both diseases can be triggered by viral infection, with RV being the most common etiological agent, followed by RSV.^4^

The introduction of molecular testing as standard laboratory practice to determine the presence RSV, influenza, and other respiratory viruses has led to a better understanding of the burden of viral LRTIs in hospitalized children. Interest in respiratory viral infections has been heightened during the COVID‐19 pandemic; however, there is scant information about the surveillance of respiratory viruses other than RSV, influenza, and severe acute respiratory syndrome coronavirus‐2 (SARS‐CoV‐2) in hospitalized paediatric patients. Viral LRTIs are not only interesting from a microbiological and clinical perspective, they also impact hospital bed occupancy rates and healthcare costs, and data on the latter can help hospital managers plan the distribution of their sources during annual epidemics. In this study, we analyse the demographic, clinical, and viral characteristics of LRTIs together with the cost of paediatric hospitalizations due to viral LRTI other than SARS‐CoV‐2 in a referral hospital in Southwestern Europe in order to improve understanding of the burden of viral LRTIs in hospitalized children.

## 2 METHODS

### 2.1 Study design and participants

This is a descriptive, observational, retrospective, longitudinal study performed from October 2012 (week 42) to December 2020 (week 50) in the Vall d'Hebron University Hospital in Barcelona (Spain), the largest hospital complex in the Catalan Health System, which is also a tertiary paediatric hospital catering for around 9,200 paediatric hospitalizations per year. The hospital has 16 PICU beds that can be increased to 20 during seasonal periods and 55 in general paediatric ward. This is the reference hospital for the population living in the northern part of Barcelona's metropolitan area, including 30, 508 inhabitants under 15 years old (2.68% of Catalan population under 15 years). Moreover, it is also the main paediatric hospital in Catalonia for patients requiring PICU care or solid organ transplant as well as for patients suffering from oncohematologic diseases or some forms of rare diseases being the population covered actually higher than the above‐mentioned.

All consecutive paediatric patients (<16 years old) with laboratory‐confirmed viral LRTI other than SARS‐CoV‐2 who required hospitalization during the study period were included. Respiratory samples for laboratory confirmation were collected within 3 days before and 2 days after admission. The reason for hospitalization was considered to be an LRTI if, according to the International Classification of Diseases ninth (ICD‐9) or tenth (ICD‐10) revision (Table S1), the main diagnosis was bronchiolitis (466, J21), CAP (480, 483–488, J12, J16‐J18, J09‐J11), wheezing, or asthma exacerbation (466, 493, J20, J45), or the main diagnosis was influenza (487, 488, J09‐J11), respiratory failure (518, J96) or neonatal infection (771, P28, P39, P96) with a secondary diagnosis of bronchiolitis, CAP, wheezing or asthma exacerbation.

Patients that met the inclusion criteria were classified in three groups according to the main clinical diagnosis: bronchiolitis—defined as the first episode of viral LRTI in an infant aged under 2 years as per our local protocol,^6^ CAP and wheezing/asthma.

### 2.2 Variables analysed

Demographical and clinical data were retrospectively gathered from structured data in each patient's electronic clinical record by the Hospital Information Systems. These data included: main and secondary diagnosis, respiratory viruses detected, age, sex, date of hospitalization and discharge, admission to the PICU, and if so, date of admission and discharge, need for respiratory support and maximum support administered (conventional oxygen, high flow nasal cannula, or non‐invasive mechanical ventilation (HFNC/NIMV), invasive mechanical ventilation (IMV) and extracorporeal membrane oxygenation (ECMO) (Table S2). All the information was uploaded to a codified database.

Hospitalization cost was based on the prices established by our hospital and calculated based on 2021 fares: €615 per day in the paediatric ward and €2,350 per day in the PICU (internal data from the Catalan Health System data). Surveillance seasons lasted from week 40 in October to week 20 in May.

### 2.3 Procedures

Respiratory tract specimens were collected within 3 days prior to admission for LRTI from all emergency department patients with symptoms of respiratory infection. Thereafter, respiratory specimens were taken within two day of admission to the paediatric ward or the PICU. Samples were processed in the hospital microbiology laboratory within 24 hours for routine testing, and within six hours for rapid detection tests for influenza viruses and RSV. From October 2012 to May 2016, detection of respiratory viruses was routinely performed by direct immunofluorescent antigen detection assay (D3 Ultra 8™ DFA Respiratory Virus Screening & Identification Kit, Diagnostic HYBRIDS, USA), which detects influenza A (FLUAV) and B (FLUBV), RSV, adenovirus (AdV), human parainfluenza viruses 1–3 (HPIV 1–3) and human metapneumovirus (HMPV). From October 2014 to November 2016, real‐time multiplex RT‐PCR—first, the Anyplex™ II RV16 Detection kit (Seegene, South Korea), which was later replaced by the Allplex™ Respiratory Panel (Seegene, South Korea)—was included for routine testing for the foregoing respiratory viruses in addition to HPIV‐4, bocavirus (BoV), enterovirus (EV), RV, and human coronaviruses (hCoV) NL63, OC43 and 229E. Rapid RSV and influenza testing during epidemics was performed using an immunofluorescent assay (Sofia RSV or Influenza FIA, Quidel, CA, USA) or a rapid molecular test (GeneXpert Flu or Flu*/*RSV XC, Cepheid, CA, USA). If the rapid test was negative, the study was extended to include other respiratory viruses that were detected using routine testing techniques.

### 2.4 Statistical analysis

Continuous variables are described as mean and standard deviation (SD) and categorical variables as frequencies and proportions. Contingency tables were used to analyse the relationship between two qualitative variables, and chi‐square tests were used to assess associations. The Kruskal Wallis test was used to analyse the relationship between quantitative and qualitative variables. A logistic regression model was used to characterize the response variables, and the results were presented as an odds ratio (OR) with the corresponding 95% confidence interval (95% CI). A multivariate logistic regression model was used to determine the risk factors associated with PICU admission. In all models, pair‐wise comparisons were performed using Tukey correction for multiple comparisons. The significance level was set at 0.05 for all tests. The results were analysed using SAS v9.4 (SAS Institute Inc., Cary, NC, USA).

## 3 RESULTS

During the study period, 29,511 children aged <16 years were admitted from the paediatric emergency department, 4,056 (13.74%) with a diagnosis of viral LRTI. The LRTI episodes corresponded to 3,325 children, of whom 1,317 (32.47%) were under 12 months of age, 1,902 (57.20%) were male, 314 (9.44%) had a history of chronic disease, and 146 (3.60%) were immunocompromised. Four (0.10%) patients died.

In total, 4,753 viruses were isolated from the 4,056 patients with viral LRTI (≥2 viruses were detected in 565 patients). Age distribution of LRTI and the viruses isolated are shown in Table 1. The most common virus causing LRTI was RSV, which was detected in 2,133 (52.59%) children, followed by RV (1,210 [29.83%]), and HMPV (341 [8.41%]). Among the 84 hCoV diagnoses, 12 corresponded to hCoV‐229E, 28 to hCoV‐NL63, and 44 to hCoV‐OC43. Of the 255 HPIV diagnoses, 39 were HPIV‐1, 9 HPIV‐2, 149 HPIV‐3 and 58 HPIV‐4. The most frequent co‐detections were RSV‐RV and AdV‐RV (83/565 and 64/565, respectively) (Table S3). Hospitalization occurred during the surveillance seasons (from week 40 in October to week 20 in May) in 3,684 (90.82%) of study cases. According to clinical diagnosis, of the 4,056 patients hospitalized for viral LRTI, 2,185 (53.87%) presented wheezing/asthma, 1,535 (37.84%) bronchiolitis, and 336 (8.28%) CAP. Three‐hundred and sixty patients with LRTIs (8.87%) required PICU admission, representing 7.89% of all patients admitted to the PICU during the study period. Overall, 333/360 (92.50%) were admitted during the surveillance seasons. One hundred and eighty children were admitted directly to the PICU from other centres. Of the four non‐surviving patients, one died due to FLUAV CAP, two due to CAP caused by RSV in children with a history of neurologic comorbidities, and one due to RSV‐related wheezing/asthma in a child with chronic lung disease.

**TABLE 1:** Age distribution of PICU patients, LRTI, and isolated viruses

	Total (column)	≤12 months	12–24 months	>24 month‐4 years	5–15 years	*p* value
Total (row), *n* (%)		1,317 (32.47%)	1,120 (27.61%)	1,117 (27.54%)	502 (12.38%)	
Number of PICU patients, *n* (%)	360	227 (63.06%)	79 (21.94%)	35 (9.72%)	19 (5.28%)	<0.0001
LRTI, *n* (%)						
Bronchiolitis	1,535	1,187 (77.33%)	348 (22.67%)	0 (0.0%)	0 (0.0%)	<0.0001
Wheezing/asthma	2,185	116 (5.31%)	712 (32.58%)	938 (42.93%)	419 (19.18%)	<0.0001
CAP	336	14 (4.17%)	91 (27.08%)	154 (45.83%)	77 (22.92%)	<0.0001
Virus, *n* (%)						
FLUAV	136	22 (16.18%)	42 (30.88%)	50 (36.76%)	22 (16.18%)	0.0009
FLUBV	53	10 (18.87%)	11 (20.75%)	19 (35.85%)	13 (24.53%)	0.0130
RSV	2,133	1,002 (46.98%)	583 (27.33%)	469 (21.99%)	79 (3.70%)	<0.0001
HMPV	341	104 (30.50%)	122 (35.78%)	92 (26.98%)	23 (6.74%)	0.0004
AdV	229	16 (6.99%)	91 (39.74%)	99 (43.23%)	23 (10.04%)	<0.0001
hCoV^a^	84	17 (20.48%)	19 (22.89%)	36 (43.37%)	11 (13.25%)	0.0097
RV	1,210	183 (15.12%)	279 (23.06%)	415 (34.30%)	333 (27.52%)	<0.0001
BoV	169	6 (3.55%)	93 (55.03%)	68 (40.24%)	2 (1.18%)	<0.0001
HPIV^a^	255	55 (21.74%)	80 (31.62%)	83 (32.81%)	35 (13.83%)	0.0041
EV	143	5 (3·50%)	50 (34.97%)	68 (47.55%)	20 (13.99%)	<0.0001

Abbreviations: AdV, adenovirus; BoV, bocavirus; CAP, community acquired pneumonia; EV, enterovirus; FLUAV, influenza A virus; FLUBV, influenza B virus; hCoV, human coronaviruses; HMPV, human metapneumovirus; HPIV 1–4, human parainfluenza viruses 1–4; LRTI, lower respiratory tract infection; PICU, paediatric intensive care unit; RSV, respiratory syncytial virus; RV, rhinovirus·.

^a^ One patient with hCoV‐229E and hCoVNL63 coinfection and 2 patients with hPIV‐3 and hPIV‐4 coinfection were only taken into account once.

Seasonal differences in the percentage of laboratory‐confirmed cases were observed in FLUAV, AdV, RSV, RV (*p* < 0.0001 for each), FLUBV (*p* = 0.0199), HMPV (*p* = 0.0006), HPIV‐3 (*p* = 0.0037), and BoV (*p* = 0.0112), especially during the 2020–2021 season when the annual FLUAV, FLUBV, RSV and HMPV epidemics were not observed, and RV and AdV were the main viruses circulating that season (Figure S1 and Table S4). Additionally, statistically significant differences were observed in the monthly trends of FLUAV, FLUBV, RSV, HMPV, AdV, RV, HPIV 3–4, and EV (*p* < 0.0001 for each) (Figure S2). During seasonal periods, RSV was the first virus to peak (in December), followed by FLUAV peaking in February, FLUBV in March, and HMPV between March and April.

Analysing the main clinical findings related to laboratory‐confirmed LRTI cases for respiratory viruses (Table 2), we found that 3,068 (75.64%) of patients with LRTIs required respiratory support, of which 2,750 (89.63%) needed conventional oxygen supply, 234 (7.63%) HFNC/NIMV, 82 (2.67%) IMV, and 2 (0.07%) ECMO. The two cases requiring ECMO were a previously healthy four year‐old girl with no disease history who presented severe pneumonia due to FLUAV‐*Streptococcus pneumoniae* co‐infection, and a 4 year old girl with severe neurologic sequelae due to a brain tumour who presented RSV CAP, which was ultimately fatal. FLUAV caused longer hospitalizations than the other viruses (7.51 ± 31.58 days, *p* = 0.0077). The highest proportion of patients needing admission to the PICU (241/2,133; 11.23%, *p* < 0.0001) and respiratory support (1,680/2,133; 78.76%, *p* < 0.0001) presented RSV infection. In contrast, RV‐related disease was milder (length of stay [LOS]: 4.04 ± 9.45 days, *p* = 0.0065; PICU admission: 6.94% of patients, *p* = 0.0057).

**TABLE 2:** Association between causative viruses and mean hospital LOS, PICU admission and respiratory support

	FLUAV	FLUBV	RSV	HMPV	AdV	hCoVs (OC‐43, 229‐E, and NL‐63)^a^	RV	BoV	hPIVs (1‐4)^a^	EV	*p* value^b^
Total	136	53	2,133	341	229	83	1,210	169	253	143	
LOSMean (SD) *p* value^c^	7.51 (31.58) *0.0077*	5.35 (3.57) *0.7361*	5.16 (12.51) *0.0184*	4.49 (3.32) *0.6164*	3.78 (3.41) *0.2088*	4.28 (4.75) *0.6521*	4.04 (9.45) *0.0065*	3.86 (5.07) *0.3489*	4.85 (6.07) *0.9864*	3.54 (2.7) *0.2298*	*=0.0126*
PICU admission, N (%) *p* value^c^	5 (3.68%) *0.0378*	4 (7.55%) *0.7272*	241 (11.23%) *<0.0001*	21 (6.16%) *0.0709*	9 (3.93%) *0.0102*	8 (9.64%) *0.7984*	84 (6.94%) *0.0057*	16 (9.47%) *0.7803*	12 (4.74%) *0.0193*	11 (7.69%) *0.6315*	*<0.0001*
Respiratory support, N (%) *p* value^c^	97 (71.32%) *0.2615*	39 (73.58%) *0.7084*	1,680 (78.76%) *<0.0001*	259 (75.95%) *0.9077*	155 (67.69%) *0.0072*	58 (69.88%) *0.2596*	905 (74.79%) *0.4485*	131 (77.51%) *0.5876*	161 (63.64%) *<0.0001*	108 (75.52%) *0.9933*	*<0.0001*
Maximum respiratory support, *p* value^c^	*0.0779*	*0.4517*	*0.0027*	*0.1533*	*0.2033*	*0.4129*	*0.2917*	*0.0139*	*0.5864*	*0.5180*	
ECMO, N (%)^d^	1 (0.74%)	0	1 (0.05%)	0	0	0	0	0	0	0	
IMV, N (%)^d^	4 (2.94%)	0	51 (2.39%)	5 (1.47%)	3 (1.31%)	1 (1.20%)	25 (2.07%)	2 (1.18%)	3 (1.19%)	2 (1.4%)	
HFNC/NIMV, N (%)^d^	4 (2.94%)	2 (3.77%)	153 (7.17%)	12 (3.52%)	6 (2.62%)	8 (9.64%)	59 (4.89%)	11 (6.51%)	5 (1.98%)	7 (4.9%)	
O_2_, N (%)^d^	88 (64.71%)	37 (69.81%)	1,475 (69.15%)	242 (71.00%)	146 (63.76%)	49 (59.04%)	821 (67.85%)	118 (69.82%)	153 (60.47%)	99 (69.23%)	

Abbreviations: AdV, adenovirus; BoV, bocavirus; ECMO, extracorporeal membrane oxygenation; EV, enterovirus; FLUAV, influenza A virus; FLUBV, influenza B virus; hCoV, human coronaviruses; HFNC/NIMV, high flow nasal oxygen/non‐invasive mechanical ventilation; HMPV, human metapneumovirus; HPIV 1–4, human parainfluenza viruses 1–4; IMV, invasive mechanical ventilation; LOS, length of hospital stay; LRTI, lower respiratory tract infection; O_2_, conventional oxygen; PICU, paediatric intensive care unit; RSV, respiratory syncytial virus; RV, rhinovirus; SD, standard deviation.

^a^ One patient with hCoV‐229E and hCoVNL63 coinfection and 2 patients with hPIV‐3 and hPIV‐4 coinfection were only taken into account once for the study of virus‐related disease.

^b^ *p* values are calculated comparing positive cases of each virus.

^c^ *p* values are calculated comparing cases positive for each virus with those that tested negative for the virus studied.

^d^ From the total of each virus.

**FIGURE 1:**
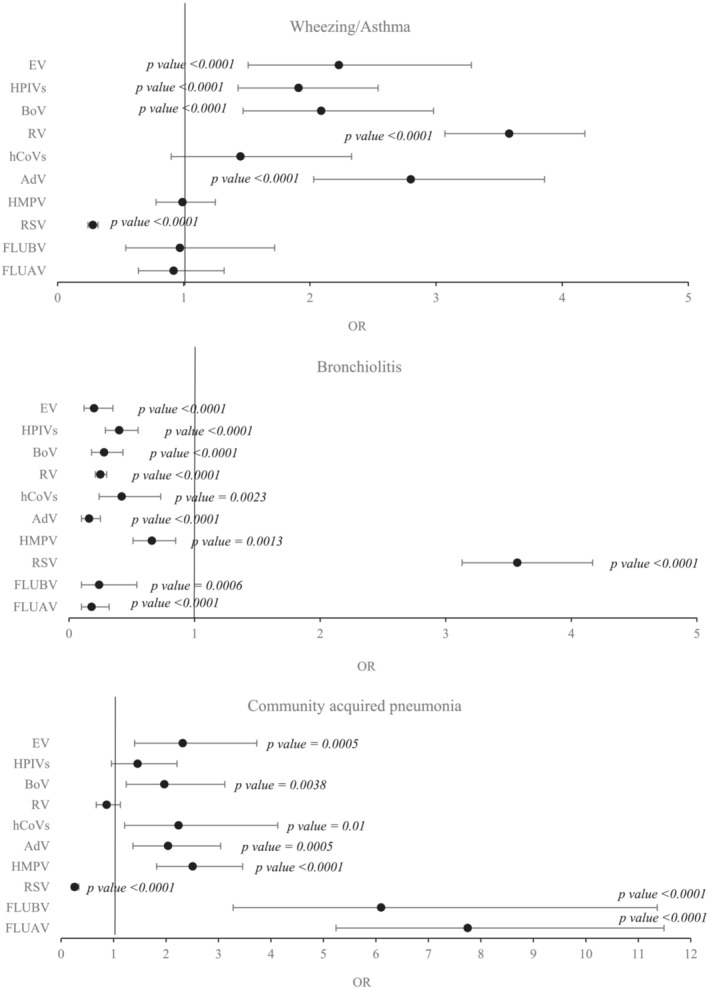
Association between causative viruses and related LRTIs (wheezing/asthma, bronchiolitis and community acquired pneumonia) expressed by OR and 95% CI. The OR is calculated by comparing the number of positive cases with those that tested negative for each virus. *p* value is only specified in statistically significant cases. AdV, adenovirus; BoV, bocavirus; 95CI, 95% confidence interval; EV, enterovirus; FLUAV, influenza A virus; FLUBV, influenza B virus; hCoVs, human coronaviruses; HMPV, human metapneumovirus; HPIVs, human parainfluenza viruses; LRTI, lower respiratory tract infection; OR, odds ratio; RSV, respiratory syncytial virus; RV, rhinovirus

To determine whether certain respiratory viruses were associated with specific LRTIs, we compared the number of patients who tested positive for each virus with those that tested negative for that virus (Figure 1). FLUAV was associated with pneumonia (OR 7.75, 95% CI 5.24–11.49; *p* < 0.0001), RSV was associated with bronchiolitis (OR 3.57, 95% CI 3.13–4.17; *p* < 0.0001), and RV caused mainly wheezing/asthma (OR 3.58, 95% CI 3.07–4.18; p < 0.0001).

We also performed multivariate analyses to identify the host factors that increased the risk of PICU admission (Table S5): male sex (*adjusted p* = 0.0048), age ≤ 12 months (*adjusted p* = 0.0002 compared with 12–24 months), history of chronic conditions (*adjusted p* < 0.0001), and bronchiolitis (*adjusted p* = 0.0004). We were unable to confirm any correlation with viruses.

Our analysis of hospitalization costs showed that during surveillance seasons, viral LRTIs accounted for 12.36% of overall bed days (17,921/144,900), 14.39% of paediatric ward bed days (15,294/106,260) and 6.79% of PICU bed days (2,627/38,640). During the entire study period, total hospitalization costs were €16,603,415 (being only €92,930 attributable to the 2020–2021 season), with a cost per patient of €4,093.54 (Table S6).

## 4 DISCUSSION

In this study, we reviewed the burden of viral LRTIs other than SARS‐CoV‐2 in children aged <16 years in one of the largest tertiary paediatric hospitals in Catalonia (Spain) from October 2012 to December 2020. Our results show that viral LRTIs are an important cause of morbidity and high healthcare costs in hospitalized children during seasonal periods, particularly in children aged <1 year, males, and in children with chronic conditions. Interestingly, 90% of study patients had no history of chronic conditions. An analysis by virus type showed that RSV was the main etiological agent.

RSV, RV, wheezing/asthma, and bronchiolitis were the most prevalent viruses and diseases during the study period; bronchiolitis increased the risk of requiring intensive care. As previously reported in the literature, and confirmed by our results, host factors such as male sex, younger age, and history of chronic conditions increase the severity of viral LRTIs, and therefore the likelihood of PICU admission.^5^, ^7^

Before the COVID‐19 pandemic, the burden of respiratory viruses was particularly high during the winter months in temperate countries such as Spain due to the annual RSV, influenza, and HMPV epidemics.^8^, ^9^, ^10^ Differences in seasonal and monthly respiratory virus trends described in our study can be explained by the interaction of environmental, host, and viral factors, in addition to the use of different laboratory diagnostic techniques.^11^, ^12^ The interaction of the foregoing factors was particularly important during the first half of the 2020–2021 season, in which the introduction of non‐pharmaceutical interventions and restrictions on international travel during the COVID‐19 pandemic prevented the spread not only of SARS‐CoV‐2, but also of other respiratory viruses, particularly RSV and influenza viruses.^8^, ^10^ Nevertheless, RV and AdV were still detected, probably because non‐pharmaceutical interventions were not as effective in these non‐enveloped viruses as in other respiratory viruses, including SARS‐CoV‐2.^13^, ^14^

In addition to the prevalence and seasonality of LRTIs, one of the most interesting factors to be considered is their burden in hospitalized children. According to the Global Burden of Diseases 2017, the burden of LRTI in children aged <5 years was lower in pre‐pandemic seasons due to improvements in hygienic and sanitary conditions and increased vaccine coverage.^15^ Nevertheless, our results show that viral LRTIs are still an important cause of morbidity in children. The few studies that have analysed the burden of LRTIs in paediatric hospitals have mainly focused on the most virulent viruses, such as RSV, influenza viruses, and HMPV.^16^, ^17^, ^18^ We, however, found that respiratory viruses other than RSV, influenza, and HMPV were responsible for a third of all cases of LRTIs, showing that the burden of these viruses is significant in terms of bed occupancy rates and healthcare costs. Overall, RSV has been described as the main cause of hospital admission and LRTI‐related deaths worldwide, followed by influenza viruses, and HMPV, particularly in children aged <1 year.^1^, ^16^, ^17^, ^18^ RSV and influenza viruses were also the main causes of morbidity and mortality in our study, as both were associated with prolonged LOS. In the case of RSV, this was probably due to the need for respiratory support and PICU admission for bronchiolitis,^19^ while in the case of influenza, it was likely due to the use of parenteral antibiotics and respiratory support for secondary bacterial infection in patients with CAP.^20^ FLUAV and RSV were associated with the four fatalities in our study, all of which occurred in children with a history of comorbidities, as previously reported.^19^, ^20^ Despite the lower number of HMPV cases, the risk of severe infection has been described as similar to that of RSV or influenza viruses, especially in children aged <1 year.^17^, ^21^ However, we observed milder outcomes in HMPV infection, probably due to the lower proportion of children aged <1 year and to differences in HMPV genotypes circulating in each region.^9^ RV, despite being the second most prevalent LRTI‐related virus, caused milder disease compared with RSV or influenza viruses, probably due to less respiratory cytokine production leading to a milder proinflammatory response.^22^ Our data showed that a high proportion of hCoV‐ and BoV‐related LRTIs required PICU admission, and a high number of BoV‐related infections required respiratory support, although this occurred mostly in cases of co‐infection with more virulent viruses, such as RSV.^23^, ^24^

Viral LRTIs are generally associated with high bed occupancy rates during surveillance seasons. In our study, the total direct health care cost due to LRTI was €16,603,415, with a per‐patient cost of €4,093.54—a considerable economic burden on the health care system, considering that the per capita budget of the Catalan Health Institute is approximately €1,293.1/year, a figure that falls far short of the real cost of these hospitalizations.^25^ In addition to direct hospital costs, the cost of treating children in primary care and secondary costs arising from parental work absenteeism, together with travel and meal expenses during the child's hospital stay, should be taken into account. This underscores the importance of research and the implementation of preventive measures to reduce morbidity, clinical costs, and bed occupancy rates. Our results suggest that these measures should focus on the prevention of RSV and influenza viruses during epidemics, especially in younger children, and should be offered to the entire paediatric population, regardless of their comorbid status. Preventive measures include influenza vaccination, which can prevent up to 50% of influenza‐related hospitalizations in children under eight years old.^26^ Other strategies currently under development are antiviral treatments based on humanized monoclonal antibodies against RSV, which can prevent 77.3% of hospital admissions for RSV‐related LRTI in children entering their first RSV season.^27^

This study has several limitations, apart from its retrospective design. Factors such as viral‐bacterial and viral‐viral co‐detections were not taken into consideration when evaluating clinical evolution, and may modify clinical severity. However, although viral‐bacterial co‐detections are known to increase disease severity, there is no conclusive evidence that viral‐viral co‐detections increase the disease burden compared with single infections.^20^, ^28^ From a microbiological point of view, we used various diagnostic techniques with varying sensitivities to detect viral species. This makes it difficult to compare data between seasons, and may have led us to underestimate the true burden of RV, BoV, hCoV, HPIV‐4 and EV, especially during the first season studied, when these viruses were not routinely detected. In addition, the use of rapid tests targeting only RSV or influenza viruses during annual epidemics may have led us to underestimate the role of viral co‐detection during the winter months. Our use of structured data allowed us to export a considerable volume of information; however, although a group of experts reviewed the disease codes, we cannot rule out the existence of disease and respiratory support coding errors. Nevertheless, large amounts of structured patient data from hospital information system are relatively easy to obtain, and will play a key role in the clinical and virological surveillance of viral LRTIs in children. The total costs may be biassed because of COVID‐19 pandemic, especially in the 2020–2021 season. Additionally, the calculated cost for this season is underrated as patients were only included until December 2020. Also, costs may have changed along the study period but we were unable to retrieve appropriate yearly data due to changes in the measurement system and economic databases so they were all calculated based on 2021 fares. Finally, despite the relevance of our findings, the single‐centre design of our study prevents us from extrapolating our results to other geographical areas. Similar studies in other regions are needed to compare findings on viral LRTIs. Aside from these limitations, our study stands out from previous research conducted in our geographical area, insofar as we describe viral LRTIs from the perspective of microbiological data, clinical outcomes, and consumption of healthcare resources.^6^, ^9^, ^29^ Finally, one of the strengths of our study is the inclusion of a large patient sample, which allowed to study the burden of viral LRTIs in hospitalized children over a long period of time.

In conclusion, viral LRTIs place a heavy burden on hospital resources, and on the healthcare system in general due to their associated morbidity. LRTI‐related mortality in our region, however, is scarce, and associated with previous chronic conditions. Continuous surveillance of LRTI will improve healthcare planning, show changes in prevalence and severity trends based on previous seasons, which may indicate the circulation of a new viral variant, and allow hospital authorities to monitor the efficacy of preventive or therapeutic measures in the paediatric population. Our experience of the recent COVID‐19 pandemic has shown that active LRTI microbiological and clinical surveillance should be a public health priority. Finally, it is essential to increase the limited number of antivirals available for influenza and RSV by developing new prophylaxis measures and drugs to treat viral LRTI. This will improve the health of the paediatric population, and reduce the consumption of hospital resources and associated costs.

## CONFLICT OF INTEREST

All authors certify that they have no affiliations with or involvement in any organization or entity with any financial interest or non‐financial interest in the subject matter or materials discussed in this manuscript.

## ETHICS STATEMENT

All procedures were performed in accordance with the ethical standards of the Vall d'Hebron Barcelona Hospital Campus Research Ethics Committee and the 1964 Helsinki Declaration and its subsequent amendments, or comparable ethical standards. The study was approved by the Vall d'Hebron Hospital Clinical Research Ethics Committee (PR[AMI]556/2020).

## AUTHOR CONTRIBUTIONS

**Jorgina Vila:** Conceptualization; data curation; investigation; methodology; project administration; resources; supervision; validation; visualization; writing‐original draft; writing‐review and editing. **Esther Lera:** Conceptualization; methodology; resources; supervision; validation; writing‐original draft; writing‐review and editing. **Cristina Andrés:** Data curation; investigation; writing‐original draft; writing‐review and editing. **Maria Piñana:** Data curation; investigation; writing‐review and editing. **Victoria Rello‐Saltor:** Investigation; writing‐original draft; writing‐review and editing. **Marc Tobeña‐Rué:** Investigation; writing‐original draft; writing‐review and editing. **Joan Balcells:** Investigation; writing‐original draft; writing‐review and editing. **Zaira Benítez‐Díaz:** Data curation; investigation; methodology; resources; software; validation; writing‐review and editing. **Marta Beatriz Aller:** Data curation; investigation; methodology; resources; software; supervision; validation; visualization; writing‐review and editing. **Rosario Muñoz:** Data curation; resources; writing‐review and editing. **Ana Vazquez:** Formal analysis; investigation; methodology; software; supervision; validation; writing‐review and editing. **Carlos Rodrigo:** Conceptualization; investigation; methodology; project administration; resources; visualization; writing‐original draft; writing‐review and editing. **Pere Soler Palacín:** Conceptualization; funding acquisition; investigation; supervision; writing‐original draft; writing‐review and editing. **Andrés Anton:** Conceptualization; data curation; funding acquisition; investigation; methodology; project administration; resources; supervision; validation; visualization; writing‐original draft; writing‐review and editing.

### PEER REVIEW

The peer review history for this article is available at https://publons.com/publon/10.1111/irv.13085.

## Supporting information


**Table S1.** Classification of lower respiratory tract infections studied according to ICD‐9 and ICD‐10 codes.
**Table S2**. Classification of respiratory support according to ICD‐9 and ICD‐10 codes.
**Table S3**. Number of viruses and virus combinations in viral co‐detections
**Table S4**. Number and percentage of monthly viruses comparing rates in the period 2012–2019 versus year 2020
**Table S5**. Host factors that increased the risk of PICU admission in the univariate and multivariate analysis
**Table S6**. Cost of the hospitalization per season (PW, PICU and total)
**Figure S1**. Seasonal number and percentage of positive cases for each virus
**Figure S2**. Monthly number and percentage of positive cases for each virus

## Data Availability

Anonymized and de‐identified participant data will be available upon request until 2 years after publication. Data will be available for researchers who provide a methodologically sound proposal. Requests may be sent to the corresponding author, and to gain access data requestors will need to sign a data access agreement.
