# Supplementary material for: The burden of non‐SARS‐CoV2 viral lower respiratory tract infections in hospitalized children in Barcelona (Spain): A long‐term, clinical, epidemiologic and economic study
Source: Influenza Other Respir Viruses. 2022 Dec 20;17(1):e13085. doi: 10.1111/irv.13085 (PMC9835422; doi:10.1111/irv.13085)

**Supplementary Material**

**Table of contents**

**Supplementary Tables**

2 Supplementary Table 1. Classification of lower respiratory tract infections studied according to ICD-9 and ICD-10 codes.

4 Supplementary Table 2. Classification of respiratory support according to ICD-9 and ICD-10 codes.

6 Supplementary Table 3. Number of viruses and virus combinations in viral co-detections

10 Supplementary Table 4. Number and percentage of monthly viruses comparing rates in the period 2012-2019 versus year 2020

13 Supplementary Table 5. Host factors that increased the risk of PICU admission in the univariate and multivariate analysis

14 Supplementary Table 6. Cost of the hospitalization per season (PW, PICU and total)

**Supplementary Figures**

15 Supplementary Figure 1. Seasonal number and percentage of positive cases for each virus

17 Supplementary Figure 2. Monthly number and percentage of positive cases for each virus

**Supplementary Table 1.** Classification of lower respiratory tract infections studied according to ICD-9 and ICD-10 codes

| Condition | ICD edition | Code | Definition |
| --- | --- | --- | --- |
| Bronchiolitis | ICD-9 | 4661 | Acute bronchiolitis |
|  |  | 46611 | Acute bronchiolitis due to respiratory syncytial virus (RSV) |
|  |  | 46619 | Acute bronchiolitis due to other infectious organisms |
|  | ICD-10 | J21.0 | Acute bronchiolitis due to respiratory syncytial virus |
|  |  | J21.1 | Acute bronchiolitis due to human metapneumovirus |
|  |  | J21.8 | Acute bronchiolitis due to other specified organisms |
|  |  | J21.9 | Acute bronchiolitis, unspecified |
| Community acquired pneumonia | ICD-9 | 480.0 | Pneumonia due to adenovirus |
|  |  | 480.1 | Pneumonia due to respiratory syncytial virus |
|  |  | 480.2 | Pneumonia due to parainfluenza virus |
|  |  | 480.8 | Pneumonia due to other virus not elsewhere classified |
|  |  | 480.9 | Viral pneumonia, unspecified |
|  |  | 483.8 | Pneumonia due to other specified organism |
|  |  | 484 | Pneumonia in infectious diseases classified elsewhere |
|  |  | 485 | Bronchopneumonia, organism unspecified |
|  |  | 486 | Pneumonia, organism unspecified |
|  |  | 487.0 | Influenza with pneumonia |
|  |  | 488.01 | Influenza due to identified avian influenza virus with pneumonia |
|  |  | 488.11 | Influenza due to identified 2009 H1N1 influenza virus with pneumonia |
|  |  | 488.81 | Influenza due to identified novel influenza A virus with pneumonia |
|  | ICD-10 | J12.0 | Adenoviral pneumonia |
|  |  | J12.1 | Respiratory syncytial virus pneumonia |
|  |  | J12.2 | Parainfluenza virus pneumonia |
|  |  | J12.3 | Human metapneumovirus pneumonia |
|  |  | J12.8 | Other viral pneumonia |
|  |  | J12.9 | Viral pneumonia, unspecified |
|  |  | J16.8 | Pneumonia due to other specified infectious organisms |
|  |  | J17* | Pneumonia in diseases classified elsewhere |
|  |  | J18.0 | Bronchopneumonia, unspecified |
|  |  | J18.1 | Lobar pneumonia, unspecified |
|  |  | J18.2 | Hypostatic pneumonia, unspecified |
|  |  | J18.8 | Other pneumonia, organism unspecified |
|  |  | J18.9 | Pneumonia, unspecified |
|  |  | J09.X1 | Influenza due to identified novel influenza A virus with pneumonia |
|  |  | J10.00 | Influenza due to other identified influenza virus with unspecified type of pneumonia |
|  |  | J10.01 | Influenza due to other identified influenza virus with the same other identified influenza virus pneumonia |
|  |  | J10.08 | Influenza due to other identified influenza virus with other specified pneumonia |
|  |  | J11.00 | Influenza due to unidentified influenza virus with unspecified type of pneumonia |
|  |  | J11.08 | Influenza due to unidentified influenza virus with specified pneumonia |
| Wheezing/Asthma | ICD-9 | 466.0 | Acute bronchitis |
|  |  | 49301 | Extrinsic asthma with status asthmaticus |
|  |  | 49302 | Extrinsic asthma with (acute) exacerbation |
|  |  | 49311 | Intrinsic asthma with status asthmaticus |
|  |  | 49312 | Intrinsic asthma with (acute) exacerbation |
|  |  | 49321 | Chronic obstructive asthma with status asthmaticus |
|  |  | 49322 | Chronic obstructive asthma with (acute) exacerbation |
|  |  | 49391 | Asthma, unspecified type, with status asthmaticus |
|  |  | 49392 | Asthma, unspecified type, with (acute) exacerbation |
|  | ICD-10 | J20.3 | Acute bronchitis due to coxsackievirus |
|  |  | J20.4 | Acute bronchitis due to parainfluenza virus |
|  |  | J20.5 | Acute bronchitis due to respiratory syncytial virus |
|  |  | J20.6 | Acute bronchitis due to rhinovirus |
|  |  | J20.7 | Acute bronchitis due to echovirus |
|  |  | J20.8 | Acute bronchitis due to other specified organisms |
|  |  | J20.9 | Acute bronchitis, unspecified |
|  |  | J45.21 | Mild intermittent asthma with (acute) exacerbation |
|  |  | J45.22 | Mild intermittent asthma with status asthmaticus |
|  |  | J45.31 | Mild persistent asthma with (acute) exacerbation |
|  |  | J45.32 | Mild persistent asthma with status asthmaticus |
|  |  | J45.41 | Moderate persistent asthma with (acute) exacerbation |
|  |  | J45.42 | Moderate persistent asthma with status asthmaticus |
|  |  | J45.51 | Severe persistent asthma with (acute) exacerbation |
|  |  | J45.51 | Severe persistent asthma with status asthmaticus |
|  |  | J45.901 | Unspecified asthma with (acute) exacerbation |
|  |  | J45.902 | Unspecified asthma with status asthmaticus |
| Influenza | ICD-9 | 4871 | Influenza with other respiratory manifestations |
|  |  | 4878 | Influenza with other manifestations |
|  |  | 48802 | Influenza due to identified avian influenza virus with other respiratory manifestations |
|  |  | 48809 | Influenza due to identified avian influenza virus with other manifestations |
|  |  | 48812 | Influenza due to identified 2009 H1N1 influenza virus with other respiratory manifestations |
|  |  | 48819 | Influenza due to identified 2009 H1N1 influenza virus with other manifestations |
|  |  | 48882 | Influenza due to identified novel influenza A virus with other respiratory manifestations |
|  | ICD-10 | J09.X2 | Influenza due to identified novel influenza A virus with other respiratory manifestations |
|  |  | J09.X9 | Influenza due to identified novel influenza A virus with other manifestations |
|  |  | J10.1 | Influenza due to other identified influenza virus with other respiratory manifestations |
|  |  | J10.89 | Influenza due to other identified influenza virus with other manifestations |
|  |  | J11.1 | Influenza due to unidentified influenza virus with other respiratory manifestations |
|  |  | J11.89 | Influenza due to unidentified influenza virus with other manifestations |
| Respiratory failure | ICD-9 | 51881 | Acute respiratory failure |
|  |  | 51882 | Other pulmonary insufficiency, not elsewhere classified |
|  |  | 51884 | Acute and chronic respiratory failure |
|  | ICD-10 | J96.00 | Acute respiratory failure |
|  |  | J96.01 | Acute respiratory failure with hypoxia |
|  |  | J96.02 | Acute respiratory failure with hypercapnia |
|  |  | J96.20 | Acute and chronic respiratory failure |
|  |  | J96.21 | Acute and chronic respiratory failure with hypoxia |
|  |  | J96.22 | Acute and chronic respiratory failure with hypercapnia |
|  |  | J96.90 | Respiratory failure, unspecified, unspecified whether with hypoxia or hypercapnia |
|  |  | J96.91 | Respiratory failure, unspecified with hypoxia |
|  |  | J96.92 | Respiratory failure, unspecified with hypercapnia |
| Infection in the newborn | ICD-9 | 771.89 | Other infections specific to the perinatal period |
|  | ICD-10 | P28.89 | Other specified respiratory conditions of newborn |
|  |  | P39.9 | Infection specific to the perinatal period, unspecified |
|  |  | P39.8 | Other specified infections specific to the perinatal period |
|  |  | P96.89 | Other specified conditions originating in the perinatal period |
| ICD: International classification of diseases | | | |

**Supplementary Table 2.** Classification of respiratory support according to ICD-9 and ICD-10 codes

| Respiratory support | ICD edition | Code | Definition |
| --- | --- | --- | --- |
| Conventional oxygen | ICD-9 | 93.96 | Other oxygen enrichment |
|  | ICD-10 | 3E0F7SF | Introduction of Other Gas into Respiratory Tract, Via Natural or Artificial Opening |
|  |  | 5A0522C | Extracorporeal Supersaturated Oxygenation, Continuous |
| High flow nasal oxygen or non-invasive mechanical ventilation | ICD-9 | 93.99 | Other respiratory procedures  Continuous negative pressure ventilation [CNP]  Postural drainage |
|  |  | 93.90 | Non-invasive mechanical ventilation  Bi-level airway pressure  BiPAP without (delivery through) endotracheal tube or tracheostomy  CPAP without (delivery through) endotracheal tube or tracheostomy  Mechanical ventilation NOS  Non-invasive positive pressure (NIPPV)  Non-invasive PPV  NPPV  That delivered by non-invasive interface:  face mask  nasal mask  nasal pillow  oral mouthpiece  oronasal mask |
|  |  | 93.91 | Intermittent positive pressure breathing [IPPB] |
|  | ICD-10 | 5A09357 | Assistance with Respiratory Ventilation, Less than 24 Consecutive Hours, Continuous Positive Airway Pressure |
|  |  | 5A09358 | Assistance with Respiratory Ventilation, Less than 24 Consecutive Hours, Intermittent Positive Airway Pressure |
|  |  | 5A09359 | Assistance with Respiratory Ventilation, Less than 24 Consecutive Hours, Continuous Negative Airway Pressure |
|  |  | 5A0935A | Assistance with Respiratory Ventilation, Less than 24 Consecutive Hours, High Nasal Flow/Velocity |
|  |  | 5A0935B | Assistance with Respiratory Ventilation, Less than 24 Consecutive Hours, Intermittent Negative Airway Pressure |
|  |  | 5A0935Z | Assistance with Respiratory Ventilation, Less than 24 Consecutive Hours |
|  |  | 5A09457 | Assistance with Respiratory Ventilation, 24-96 Consecutive Hours, Continuous Positive Airway Pressure |
|  |  | 5A09458 | Assistance with Respiratory Ventilation, 24-96 Consecutive Hours, Intermittent Positive Airway Pressure |
|  |  | 5A09459 | Assistance with Respiratory Ventilation, 24-96 Consecutive Hours, Continuous Negative Airway Pressure |
|  |  | 5A0945A | Assistance with Respiratory Ventilation, 24-96 Consecutive Hours, High Nasal Flow/Velocity |
| Invasive mechanical ventilation | ICD-9 | 96.70 | Other continuous invasive mechanical ventilation |
|  |  | 96.71 | Continuous invasive mechanical ventilation for less than 96 consecutive hours |
|  |  | 96.72 | Continuous invasive mechanical ventilation for 96 consecutive hours or more |
|  | ICD-10 | 5A19054 | Respiratory Ventilation, Single, Nonmechanical |
|  |  | 5A1935Z | Respiratory Ventilation, Less than 24 Consecutive Hours |
|  |  | 5A1945Z | Respiratory Ventilation, 24-96 Consecutive Hours |
|  |  | 5A1955Z | Respiratory Ventilation, Greater than 96 Consecutive Hours |
| ECMO | ICD-9 | 39.65 | Extracorporeal membrane oxygenation [ECMO] |
|  | ICD-10 | 5A1522F | Extracorporeal Oxygenation, Membrane, Central |
|  |  | 5A1522G | Extracorporeal Oxygenation, Membrane, Peripheral Veno-arterial |
|  |  | 5A1522H | Extracorporeal Oxygenation, Membrane, Peripheral Veno-venous |
| ECMO: Extracorporeal membrane oxygenation; ICD: International classification of diseases | | | |

**Supplementary Table 3.** Number of viruses and virus combinations in viral co-detections

| Co-detections |  | N | % |
| --- | --- | --- | --- |
| Two viruses (N 445) | AdV + BoV | 8 | 1·80 |
|  | AdV + hCoV-OC43 | 1 | 0·22 |
|  | AdV + EV | 4 | 0·90 |
|  | AdV + HPIV-1 | 1 | 0·22 |
|  | AdV + HPIV-3 | 4 | 0·90 |
|  | AdV + RV | 64 | 14·38 |
|  | BoV + EV | 2 | 0·45 |
|  | BoV + HPIV-1 | 2 | 0·45 |
|  | BoV + HPIV-3 | 1 | 0·22 |
|  | BoV + HPIV-4 | 4 | 0·90 |
|  | hCoV-229E + hCoV-NL63 | 1 | 0·22 |
|  | hCoV-229E + RV | 5 | 1·12 |
|  | hCoV-NL63 + BoV | 2 | 0·45 |
|  | hCoV-NL63 + EV | 1 | 0·22 |
|  | hCoV-NL63 + HPIV-3 | 1 | 0·22 |
|  | hCoV-NL63 + RV | 7 | 1·57 |
|  | hCoV-OC43 + BoV | 2 | 0·45 |
|  | hCoV-OC43 + EV | 1 | 0·22 |
|  | hCoV-OC43 + RV | 10 | 2·25 |
|  | FLUAV + AdV | 1 | 0·22 |
|  | FLUAV + EV | 1 | 0·22 |
|  | FLUAV + HMPV | 1 | 0·22 |
|  | FLUAV + RV | 2 | 0·45 |
|  | FLUAV + RSV | 6 | 1·35 |
|  | FLUBV + RV | 1 | 0·22 |
|  | FLUBV + RSV | 1 | 0·22 |
|  | HMPV + AdV | 6 | 1·35 |
|  | HMPV + BoV | 8 | 1·80 |
|  | HMPV + hCoV-229E | 1 | 0·22 |
|  | HMPV + hCoV-NL63 | 2 | 0·45 |
|  | HMPV + hCoV-OC43 | 3 | 0·67 |
|  | HMPV + EV | 6 | 1·35 |
|  | HMPV + HPIV-3 | 1 | 0·22 |
|  | HMPV + HPIV-4 | 1 | 0·22 |
|  | HMPV + RV | 37 | 8·31 |
|  | HPIV-1 + EV | 1 | 0·22 |
|  | HPIV-2 + EV | 1 | 0·22 |
|  | HPIV-3 + EV | 2 | 0·45 |
|  | HPIV-3 + HPIV-4 | 2 | 0·45 |
|  | HPIV-4 + EV | 4 | 0·90 |
|  | RV + BoV | 40 | 8·99 |
|  | RV + EV | 25 | 5·62 |
|  | RV + HPIV-1 | 6 | 1·35 |
|  | RV + HPIV-2 | 2 | 0·45 |
|  | RV + HPIV-3 | 19 | 4·27 |
|  | RV + HPIV-4 | 6 | 1·35 |
|  | RSV + AdV | 11 | 2·47 |
|  | RSV + BoV | 13 | 2·92 |
|  | RSV + hCoV-229E | 1 | 0·22 |
|  | RSV + hCoV-NL63 | 4 | 0·90 |
|  | RSV + hCoV-OC43 | 7 | 1·57 |
|  | RSV + EV | 8 | 1·80 |
|  | RSV + MPNV | 6 | 1·35 |
|  | RSV + HPIV-2 | 1 | 0·22 |
|  | RSV + HPIV-3 | 3 | 0·67 |
|  | RSV + HPIV-4 | 1 | 0·22 |
|  | RSV + RV | 83 | 18·65 |
| Three viruses (N 108) | AdV + BoV + EV | 1 | 0·93 |
|  | AdV + hCoV-229E + RV | 1 | 0·93 |
|  | AdV + hCoV-OC43 + RV | 3 | 2·78 |
|  | AdV + HPIV-3 + EV | 1 | 0·93 |
|  | AdV + RV + BoV | 7 | 6·48 |
|  | AdV + RV + EV | 9 | 8·33 |
|  | AdV + RV + HPIV-1 | 1 | 0·93 |
|  | AdV + RV + HPIV-3 | 3 | 2·78 |
|  | AdV + RV + HPIV-4 | 1 | 0·93 |
|  | BoV + HPIV-3 + EV | 1 | 0·93 |
|  | hCoV-NL63 + RV + BoV | 1 | 0·93 |
|  | hCoV-NL63 + RV + HPIV-4 | 1 | 0·93 |
|  | hCoV-OC43 + BoV + EV | 1 | 0·93 |
|  | hCoV-OC43 + RV + HPIV-1 | 1 | 0·93 |
|  | FLUAV + AdV + RV | 1 | 0·93 |
|  | FLUAV + BoV + RV | 1 | 0·93 |
|  | FLUAV + hCoV-229E + RV | 1 | 0·93 |
|  | FLUAV + HMPV + EV | 1 | 0·93 |
|  | FLUAV + RSV + RV | 1 | 0·93 |
|  | FLUBV + RSV + RV | 2 | 1·85 |
|  | HMPV + BoV + EV | 2 | 1·85 |
|  | HMPV + hCoV-NL63+ RV | 1 | 0·93 |
|  | HMPV + RV + BoV | 2 | 1·85 |
|  | HMPV + RV + EV | 2 | 1·85 |
|  | HMPV + RV + HPIV-2 | 1 | 0·93 |
|  | HMPV + RV + HPIV-3 | 2 | 1·85 |
|  | RV + BoV + EV | 7 | 6·48 |
|  | RV + BoV + HPIV-3 | 3 | 2·78 |
|  | RV + HPIV-1+ EV | 1 | 0·93 |
|  | RV + HPIV-2+ EV | 1 | 0·93 |
|  | RV + HPIV-3+ EV | 5 | 4·63 |
|  | RV + HPIV-4+ EV | 1 | 0·93 |
|  | RSV + AdV + BoV | 5 | 4·63 |
|  | RSV + AdV + hCoV-OC43 | 2 | 1·85 |
|  | RSV + AdV + EV | 1 | 0·93 |
|  | RSV + AdV + RV | 4 | 3·70 |
|  | RSV + BoV + EV | 3 | 2·78 |
|  | RSV + BoV + HPIV-3 | 1 | 0·93 |
|  | RSV + hCoV-229E + BoV | 1 | 0·93 |
|  | RSV + hCoV-NL63+ HPIV-1 | 1 | 0·93 |
|  | RSV + hCoV-NL63+ RV | 1 | 0·93 |
|  | RSV + hCoV-OC43+ RV | 1 | 0·93 |
|  | RSV + HMPV + BoV | 1 | 0·93 |
|  | RSV + HMPV + EV | 1 | 0·93 |
|  | RSV + MPNV + RV | 1 | 0·93 |
|  | RSV + HPIV-4+ EV | 1 | 0·93 |
|  | RSV + RV + BoV | 3 | 2·78 |
|  | RSV + RV + EV | 7 | 6·48 |
|  | RSV + RV + HPIV-1 | 1 | 0·93 |
|  | RSV + RV + HPIV-3 | 1 | 0·93 |
|  | RSV + RV + HPIV-4 | 5 | 4·63 |
| Four viruses (N 12) | AdV + RV + BoV + EV | 3 | 25·00 |
|  | AdV + RV + BoV + HPIV-3 | 1 | 8·33 |
|  | HMPV + AdV + hCoV-NL63 + BoV | 1 | 8·33 |
|  | HMPV + AdV + RV + BoV | 1 | 8·33 |
|  | RSV + AdV + BoV + EV | 1 | 8·33 |
|  | RSV + AdV + RV + BoV | 2 | 16·67 |
|  | RSV + AdV + RV + EV | 1 | 8·33 |
|  | RSV + AdV + RV + HPIV-1 | 1 | 8·33 |
|  | RSV + hCoV-OC43 + BoV + EV | 1 | 8·33 |
| AdV: adenovirus; BoV: bocavirus; EV: enterovirus; FLUAV: influenza A virus; FLUBV: influenza B virus; hCoV: human coronaviruses; HMPV: human metapneumovirus; HPIV: human parainfluenza viruses; RSV: respiratory syncytial virus; RV: rhinovirus. | | | |

**Supplementary Table 4.** Number and percentage of monthly viruses comparing rates in the period 2012-2019 versus year 2020

| Virus | Year | Jan | Feb | Mar | Apr | May | June | July | Aug | Sept | Oct | Nov | Dec | *p value†* |
| --- | --- | --- | --- | --- | --- | --- | --- | --- | --- | --- | --- | --- | --- | --- |
| TOTAL | 2012-2019 | 510 | 287 | 298 | 191 | 140 | 114 | 32 | 21 | 141 | 208 | 638 | 1,265 |  |
|  | 2020 | 68 | 45 | 26 | 1 | 0 | 0 | 0 | 4 | 14 | 31 | 17 | 5 |  |
| FLUAV  N (%) | 2012-2019 | 33 (6.47) | 47 (16.38) | 24 (8.05) | 2 (1.05) | 0 | 1 (0.88) | 0 | 0 | 0 | 0 | 0 | 15 (1.19) | *0.0156* |
|  | 2020 | 15 (1.19) | 8 (17.78) | 3 (11.54) | 0 | 0 | 0 | 0 | 0 | 0 | 0 | 0 | 0 |  |
| FLUBV  N (%) | 2012-2019 | 8 (1.57) | 9 (3.14) | 19 (6.38) | 0 | 0 | 0 | 0 | 0 | 0 | 0 | 0 | 7 (0.55) | *0.0006* |
|  | 2020 | 2 (2.94) | 5 (11.11) | 3 (11.54) | 0 | 0 | 0 | 0 | 0 | 0 | 0 | 0 | 0 |  |
| RSV  N (%) | 2012-2019 | 359 (70.39) | 91 (31.71) | 39 (13.09) | 11 (5.76) | 5 (3.57) | 6 (5.26) | 3 (9.38) | 2 (9.52) | 1 (0.71) | 30 (14.42) | 420 (65.83) | 1,107 (87.51) | *<0.0001* |
|  | 2020 | 48 (70.59) | 9 (20) | 2 (7.69) | 0 | 0 | 0 | 0 | 0 | 0 | 0 | 0 | 0 |  |
| HMPV | 2012-2019 | 15 (2.94) | 44 (15.33) | 117 (39.26) | 72 (37.70) | 24 (17.14) | 12 (10.53) | 2 (6.25) | 1 (4.76) | 5 (3.55) | 1 (0.48) | 10 (1.57) | 20 (1.58) | *0.9478* |
|  | 2020 | 3 (4.41) | 4 (8.89) | 10 (38.46) | 0 | 1 (25) | 0 | 0 | 0 | 0 | 0 | 0 | 0 |  |
| AdV | 2012-2019 | 15 (2.94) | 21 (7.32) | 29 (9.73) | 15 (7.85) | 16 (11.43) | 16 (14.04) | 3 (9.38) | 1 (4.76) | 10 (7.09) | 15 (7.21) | 36 (5.64) | 40 (3.16) | *0.9790* |
|  | 2020 | 5 (7.35) | 0 | 0 | 0 | 0 | 0 | 0 | 0 | 0 | 4 (12.90) | 3 (17.65) | 0 |  |
| hCoV-229E | 2012-2019 | 4 (0.78) | 2 (0.70) | 3 (1.01) | 0 | 0 | 0 | 0 | 0 | 0 | 0 | 0 | 3 (0.24) | *‡* |
|  | 2020 | 0 | 0 | 0 | 0 | 0 | 0 | 0 | 0 | 0 | 0 | 0 | 0 |  |
| hCoV-NL63 | 2012-2019 | 6 (1.18) | 4 (1.39) | 6 (2.01) | 0 | 0 | 1 (0.88) | 0 | 0 | 0 | 1 (0.48) | 3 (0.47) | 5 (0.40) | *‡* |
|  | 2020 | 2 | 0 | 0 | 0 | 0 | 0 | 0 | 0 | 0 | 0 | 0 | 0 |  |
| hCoV-OC43 | 2012-2019 | 3 (0.59) | 3 (1.05) | 1 (0.34) | 5 (2.62) | 1 (0.71) | 1 (0.88) | 0 | 0 | 3 (2.13) | 1 (0.48) | 7 (1.10) | 10 (0.79) | *0.0005* |
|  | 2020 | 5 (7.35) | 4 (8.89) | 0 | 0 | 0 | 0 | 0 | 0 | 0 | 0 | 0 | 0 |  |
| RV | 2012-2019 | 79 (15.49) | 85 (29.62) | 90 (30.20) | 96 (50.26) | 81 (57.86) | 48 (42.11) | 12 (37.50) | 12 (57.14) | 113 (80.14) | 146 (70.19) | 201 (31.50) | 136 (10.75) | *<0.0001* |
|  | 2020 | 15 (22.06) | 17 (37.78) | 8 (30.77) | 0 | 0 | 0 | 0 | 4 (100) | 14 (100) | 31 (100) | 17 (100) | 5 (100) |  |
| BoV | 2012-2019 | 19 (3.73) | 13 (4.53) | 12 (4.03) | 7 (3.66) | 5 (3.57) | 4 (3.51) | 0 | 3 (14.29) | 3 (2.13) | 12 (5.77) | 35 (5.49) | 48 (3.79) | *0.7825* |
|  | 2020 | 1 (1.47) | 3 (6.67) | 2 (7.69) | 1 (100) | 0 | 0 | 0 | 0 | 0 | 0 | 0 | 1 (20) |  |
| HPIV-1 | 2012-2019 | 1 (0.20) | 0 | 0 | 0 | 0 | 1 (0.88) | 2 (6.25) | 1 (4.76) | 6 (4.26) | 14 (6.73) | 14 (2.19) | 0 | *‡* |
|  | 2020 | 0 | 0 | 0 | 0 | 0 | 0 | 0 | 0 | 0 | 0 | 0 | 0 |  |
| HPIV-2 | 2012-2019 | 0 | 0 | 1 (0.34) | 0 | 0 | 0 | 0 | 0 | 0 | 1 (0.48) | 5 (0.78) | 1 (0.08) | *‡* |
|  | 2020 | 0 | 1 (2.22) | 0 | 0 | 0 | 0 | 0 | 0 | 0 | 0 | 0 | 0 |  |
| HPIV-3 | 2012-2019 | 6 (1.18) | 6 (2.09) | 15 (5.03) | 26 (13.61) | 24 (17.14) | 33 (28.95) | 7 (21.88) | 2 (9.52) | 6 (4.26) | 5 (2.40) | 10 (1.57) | 9 (0.71) | *‡* |
|  | 2020 | 0 | 0 | 0 | 0 | 0 | 0 | 0 | 0 | 0 | 0 | 0 | 0 |  |
| HPIV-4 | 2012-2019 | 1 (0.20) | 1 (0.35) | 0 | 2 (1.05) | 0 | 2 (1.75) | 0 | 4 (19.05) | 1 (0.71) | 12 (5.77) | 22 (3.45) | 8 (0.63) | *‡* |
|  | 2020 | 3 (4.41) | 1 (2.22) | 1 (3.85) | 0 | 0 | 0 | 0 | 0 | 0 | 0 | 0 | 0 |  |
| EV | 2012-2019 | 1 (0.20) | 1 (0.35) | 11 (3.69) | 8 (4.19) | 17 (12.14) | 15 (13.16) | 5 (15.63) | 2 (9.52) | 19 (13.48) | 9 (4.33) | 28 (4.39) | 21 (1.66) | *‡* |
|  | 2020 | 0 | 3 (6.67) | 3 (11.54) | 0 | 0 | 0 | 0 | 0 | 0 | 0 | 0 | 0 |  |

AdV: adenovirus; Apr: April; Aug: August; BoV: bocavirus; Dec: December; EV: enterovirus; Feb: February; FLUAV: influenza A virus; FLUBV: influenza B virus; hCoV: human coronavirus; HMPV: human metapneumovirus; HPIV 1-4: human parainfluenza viruses 1-4; Jan: January; Mar: March; Nov: November; Oct: October; RSV: respiratory syncytial virus; RV: rhinovirus; Sept: September

**^†^**p values were calculated by comparing the average monthly cases in the period 2012-2019 versus 2020

*^‡^*p values were not calculated due to the small sample size

**Supplementary Table 5.** Host factors that increased the risk of PICU admission in the univariate and multivariate analysis

|  | N (%) | OR (95CI) | *p-value* | aOR (95CI) | *ap-value* |
| --- | --- | --- | --- | --- | --- |
| Male sex | 233 (64.72) | 1.42 (1.13-1.79) | *0*.*0027* | 1.40 (1.11-1.78) | *0*.*0048* |
| Age   - ≤12 months - 12-24 months - 2-4 years - 5-15 years | 227 (63.06)  79 (21.94)  35 (9.72)  19 (5.28) | REF^†^  2.74 (1.91-3.93)  6.46 (3.98-10.49)  5.28 (2.79-9.98) | *<0*.*0001*  *<0*.*0001*  *<0*.*0001* | REF^†^  1.93 (1.37-2.71)  4.14 (2.50-6.85)  3.84 (2.08-7.11) | *0*.*0002*  *<0*.*0001*  *<0*.*0001* |
| Comorbidities | 70 (19.44) | 2.22 (1.65-2.97) | *<0*.*0001* | 3.28 (2.39-4.50) | *<0*.*0001* |
| Disease   - Bronchiolitis - Wheezing/Asthma - CAP | 251 (69.72)  89 (24.72)  20 (5.56) | 4.31 (3·40-5·46)^‡^  0.25 (0.19-0.32)^‡^  0.62 (0.39-1.00)^‡^ | *<0*.*0001*  *<0*.*0001*  *0*.*0538* | REF^†^  2.12 (1.34-3.37)  1.28 (0.63-2.61) | *0*.*0004*  *0*.*68* |

aOR: adjusted odds ratio; ap-value: p-value of multivariate logistic regression, CAP: community acquired pneumonia; 95CI: 95% confidence interval; OR: odds ratio; PICU: paediatric intensive care unit; REF: reference. ^†^The OR and aOR are calculated by comparing ≤ 12 months with each age range or bronchiolitis with each disease. ^‡^The OR is calculated by comparing the number of cases with the disease with those without the disease.

**Supplementary Table 6**. Cost of the hospitalization per season (PW, PICU and total)

| Season | Total PW cost (€) | Total PICU cost (€) | Total (€) |
| --- | --- | --- | --- |
| 2012-2013 | 1,025,820 | 312,550 | 1,338,370 |
| 2013-2014 | 851,160 | 279,650 | 1,130,810 |
| 2014-2015 | 1,215,855 | 904,750 | 2,120,605 |
| 2015-2016 | 1,110,690 | 1,440,550 | 2,551,240 |
| 2016-2017 | 1,495,680 | 881,250 | 2,376,930 |
| 2017-2018 | 1,586,700 | 1,257,250 | 2,843,950 |
| 2018-2019 | 1,172,805 | 575,750 | 1,748,555 |
| 2019-2020 | 865,920 | 509,950 | 1,375,870 |
| 2020-2021 | 81,180 | 11,750 | 92,930 |

PICU: Paediatric intensive care unit; PW: Paediatric ward

**Supplementary Figure 1.** Seasonal number and percentage of positive cases for each virus. The percentage of positive cases is calculated by dividing the number of patients positive for each virus per season by the total of positive patients per season. *p values* are specified in statistically significant cases. AdV: adenovirus; BoV: bocavirus; EV: enterovirus; FLUAV: influenza A virus; FLUBV: influenza B virus; hCoV: human coronavirus; HMPV: human metapneumovirus; HPIV 1-4: human parainfluenza viruses 1-4; RSV: respiratory syncytial virus; RV: rhinovirus.


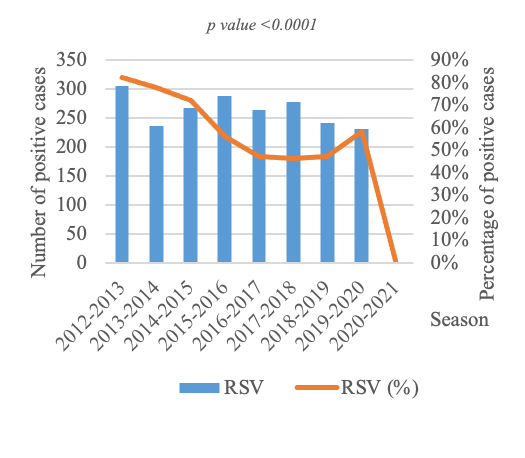

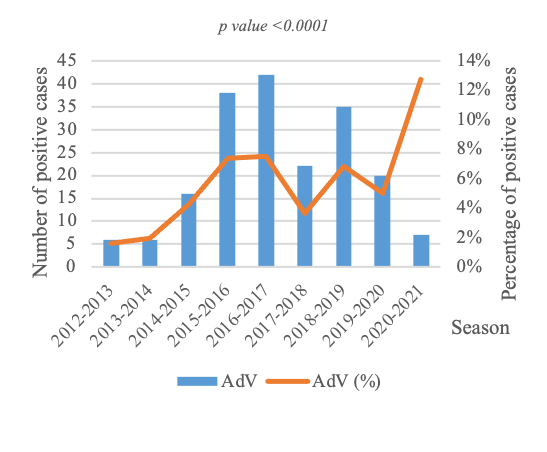

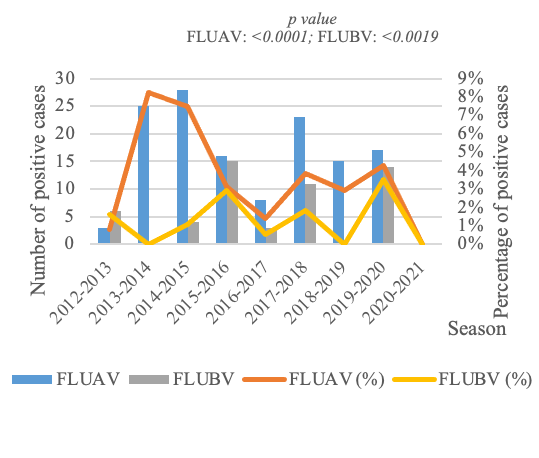

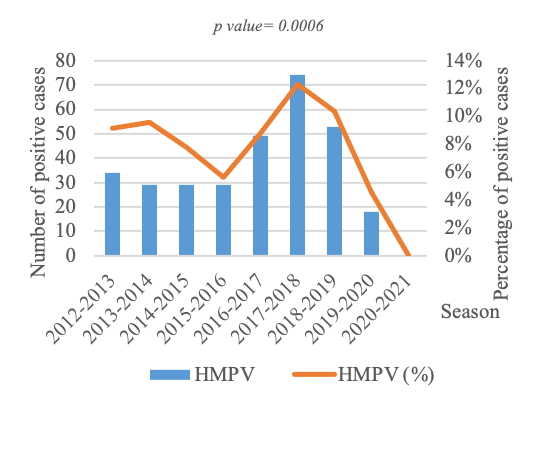

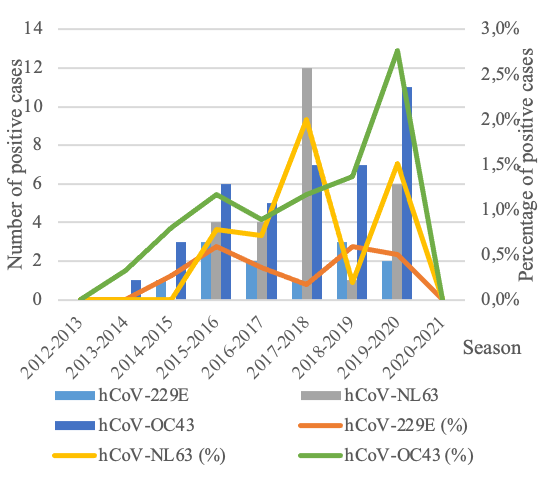

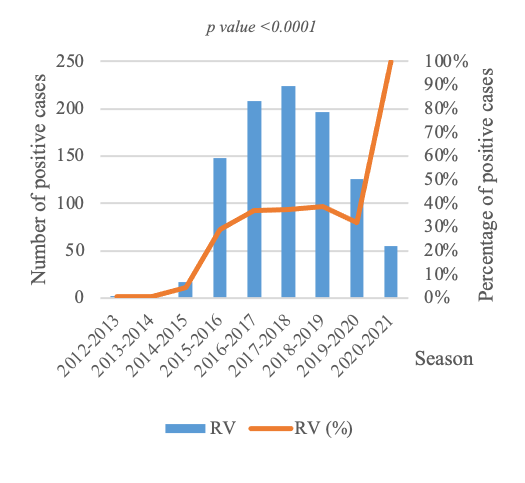

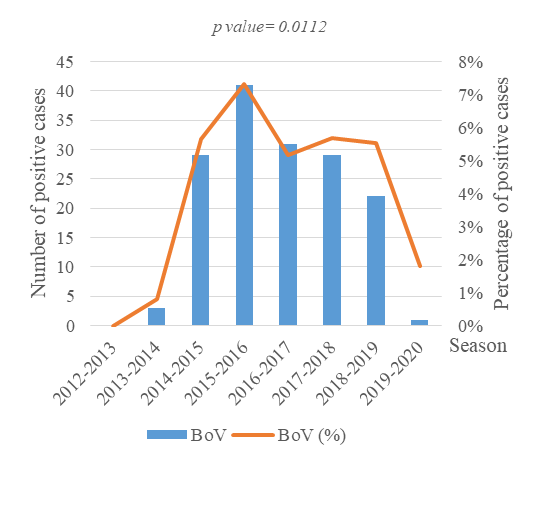

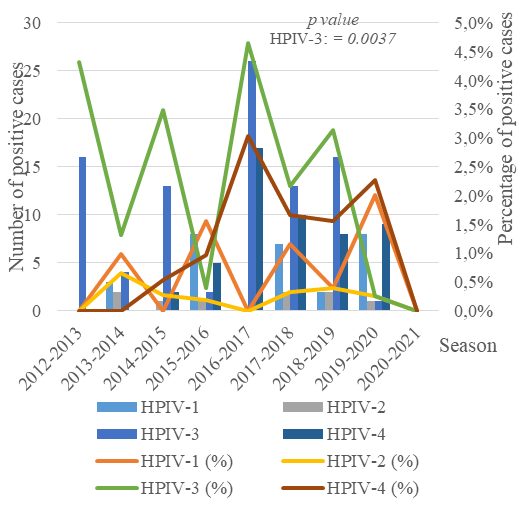

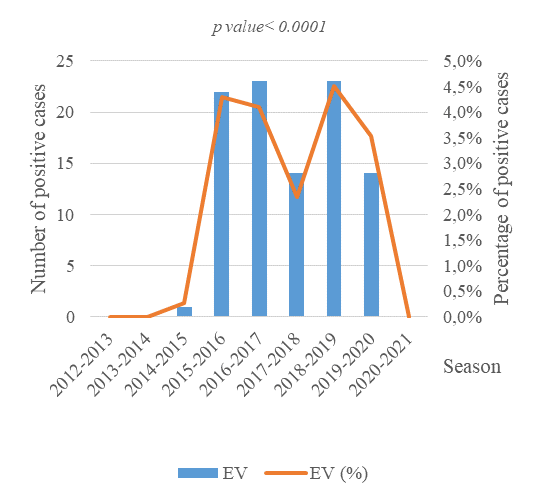


**Supplementary figure 2**. Monthly number and percentage of positive cases for each virus. The percentage of positive cases is calculated by dividing the number of patients positive for each virus per month by the total of positive patients per month. *p values* are specified in statistically significant cases. AdV: adenovirus; BoV: bocavirus; EV: enterovirus; FLUAV: influenza A virus; FLUBV: influenza B virus; hCoV: human coronavirus; HMPV: human metapneumovirus; HPIV 1-4: human parainfluenza viruses 1-4; RSV: respiratory syncytial virus; RV: rhinovirus


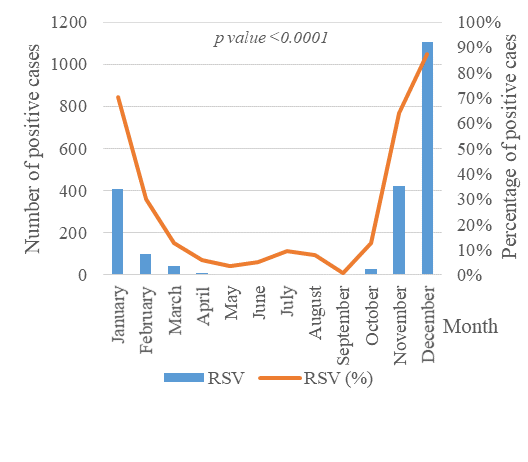

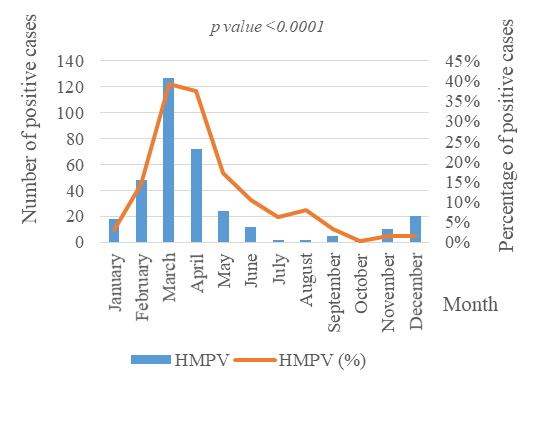

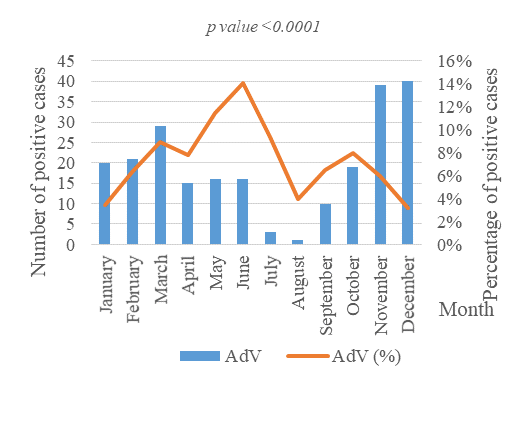

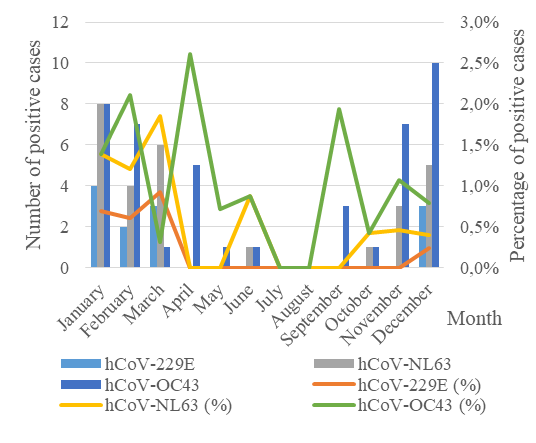

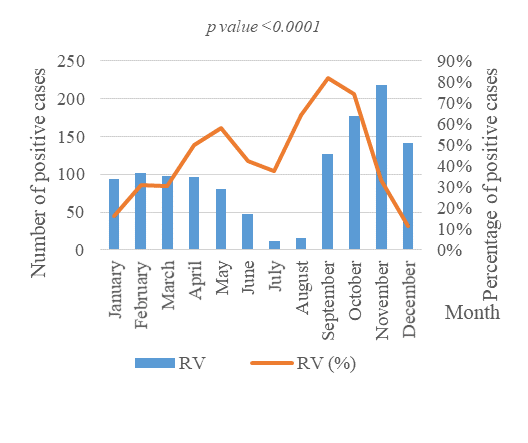

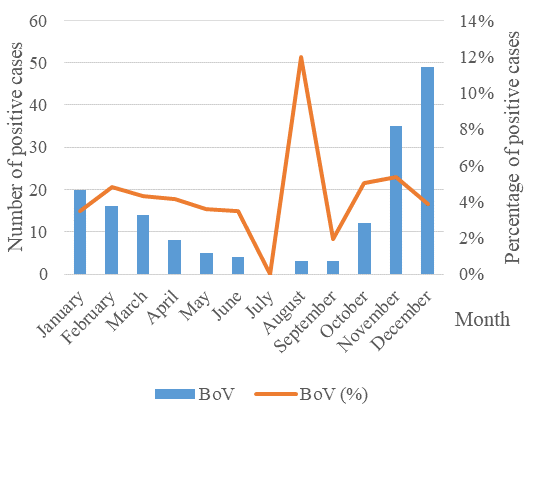

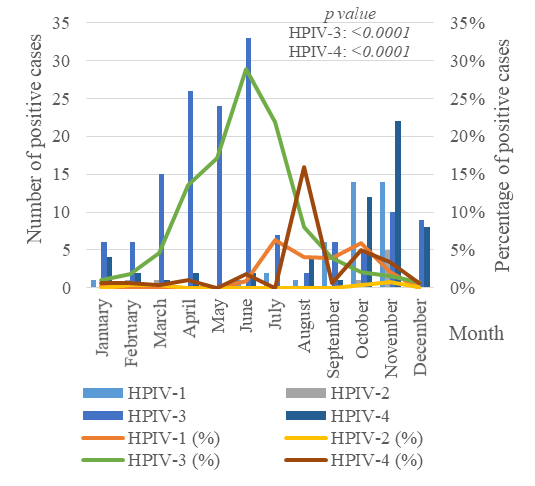

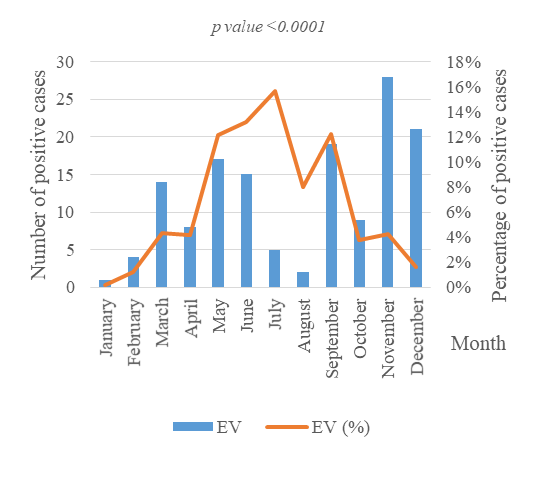

Supplement: Supplementary file 1 — Table S1. Classification of lower respiratory tract infections studied according to ICD‐9 and ICD‐10 codes. Table S2. Classification of respiratory support according to ICD‐9 and ICD‐10 codes. Table S3. Number of viruses and virus combinations in viral co‐detections Table S4. Number and percentage of monthly viruses comparing rates in the period 2012–2019 versus year 2020 Table S5. Host factors that increased the risk of PICU admission in the univariate and multivariate analysis Table S6. Cost of the hospitalization per season (PW, PICU and total) Figure S1. Seasonal number and percentage of positive cases for each virus Figure S2. Monthly number and percentage of positive cases for each virus [file IRV-17-e13085-s001.docx]
